# The perceived and/or received peer support needs of unpaid cancer caregivers residing in rural and remote areas: A systematic review

**DOI:** 10.1017/S1478951525101107

**Published:** 2025-12-12

**Authors:** Mehrnaz Keramatikerman, Bianca Viljoen, Snezana Stolic, Leah East

**Affiliations:** 1 School of Nursing and Midwifery, University of Southern Queensland, Toowoomba, QLD, Australia; 2 School of Nursing, University of Tasmania, Hobart, TAS, Australia; 3 Viertel Cancer Research Centre, Cancer Council Queensland, Brisbane, QLD, Australia; 4 School of Nursing and Midwifery, University of Southern Queensland, Ipswich, QLD, Australia; 5 Faculty of Medicine and Health, School of Health, University of New England, Armidale, NSW, Australia; 6 Centre for Health Research, University of Southern Queensland (UniSQ), Toowoomba, QLD, Australia

**Keywords:** Systematic review, cancer, unpaid caregivers, peer support, rural and remote

## Abstract

**Objective:**

Unpaid cancer caregivers (UCCs) are the primary caretakers of individuals with cancer, often shouldering caregiver responsibilities without prior preparation, which leads to a sense of isolation, particularly in remote and rural areas where healthcare access is challenging. Thus, this systematic review aimed to explore the perceived and/or received peer support needs of UCCs residing in rural and remote areas with a specific focus on informational, practical, and emotional needs.

**Method:**

Seven databases (CINAHL, ScienceDirect, PUBMED/MEDLINE, PROQUEST, Web of Science, Scopus, and Informit) were searched from 2004 to 2024. Peer-reviewed qualitative, quantitative, and mixed-method studies published in English were considered for this review. Data were extracted using the Joanna Briggs Institute System for Unified Management, Assessment, and Review of Information and presented using the Preferred Reporting Items for Systematic Reviews and Meta-Analyses flow diagram. Quality and bias were assessed with the Mixed Methods Appraisal Tool.

**Results:**

In total, 8 primary studies were included: 4 qualitative, 2 mixed methods, 1 cross-sectional, and 1 prospective survey. Four themes were identified: (1) Emotional, practical, and informational unmet needs; (2) Lack of peer support on the physical and emotional well-being of UCCs in remote and rural areas; (3) Lack of supportive services in remote and rural areas; and (4) Access to flexible peer support.

**Significance of results:**

This review revealed the unique unmet needs of UCCs in remote and rural areas, where a lack of reliable and accessible resources adversely leads to impaired UCCs’ overall well-being. Addressing these unmet needs is essential to enhance the support system for UCCs living in such regions. By identifying the gaps, the review underscores the need for developing a peer support model tailored to the specific needs of UCCs in rural and remote communities.

## Background

The incidence of cancer is rapidly growing and will affect over 35 million people globally by the end of 2050 (World Health Organization [WHO] 2024). The growth in cancer incidences can be attributed to the persistent increase in population density and an ageing population (Australian Institute of Health Welfare [AIWH] 2023). In Australia, cancer is known as one of the major contributors to mortality, accounting for 3 out of 10 deaths in 2023 (AIWH 2023). Although there have been many advances in early cancer diagnosis and an increase in survivorship during recent decades, the number of deaths due to cancer is constantly rising (Kokkonen et al. 2019).

As individuals are diagnosed with cancer and undergo medical treatment, their physical, emotional, financial, social, and psychological aspects of life are adversely impacted (Catona et al. 2022; Eyni et al. 2024; Frambes et al. 2018; Hamilton and Kroska 2019). As a result, individuals rely on unpaid caregivers’ support (Cui et al. 2023; Fenton et al. 2022). Unpaid cancer caregivers (UCCs) are considered friends and family members of individuals with cancer, who have an intimate relationship and provide care and support (Bahrami and Nasiri 2024; De Padova et al. 2019). UCCs often take on various responsibilities, from daily care such as cooking and bathing to more complex tasks such as administering medication and wound care (Bining et al. 2022; Geng et al. 2018; Stadelmaier et al. 2022). UCCs may attend regular hospital visits and take on a pivotal role in end-of-life decision-making and symptom management, particularly for loved ones with advanced stages of cancer (Alam et al. 2020; Bining et al. 2022). Moreover, UCCs’ contribution to caregiving has assisted in addressing healthcare workforce shortages and has saved an estimated $80 billion in patient-related care costs annually in Australia (Bajwah et al. 2020; Carers Australia 2020).

Cancer caregiving can have positive impacts on UCCs such as personal growth and satisfaction; however, they often feel unprepared to acquire the burden of cancer-related responsibilities (McDonald et al. 2018; Mooney et al. 2024; Too et al. 2023). This is because UCCs perform individual tasks without any previous formal training (Mollica et al. 2017; Wen et al. 2021). Literature has suggested that UCCs spend more than 40 h per week providing care for their loved ones, which can increase to 66 h in the final year of cancer (Booker et al. 2021; Xiang et al. 2022). Due to the strong emotional bond between UCCs and the individuals diagnosed with cancer, UCCs often give a high priority to their loved one’s needs and ignore their own (Lee et al. 2016; Hashemi et al. 2018) potentially leading to isolation and feelings of helplessness (Mishra et al. 2020). As a result, UCCs often experience high levels of depression, anxiety, and emotional distress due to unmet supportive needs while caring for individuals with cancer (Wang 2021; Morgan et al. 2022). Therefore, it is important to facilitate access to informational, practical, and emotional support for UCCs to promote their well-being while providing care for individuals diagnosed with cancer (Li et al. 2021).

Social support is recognised as a significant factor in enhancing individuals’ well-being. It mitigates the adverse effects of stressful circumstances and provides caregivers with essential informational, emotional, and practical assistance at every stage of the cancer continuum (Leggett et al. 2021). Social support can be assumed as either perceived or received support by individuals (Leggett et al. 2021). Perceived social support can be described as an individual’s subjective assessment of the availability of support when needed, whereas received social support refers to practical assistance provided to an individual (Gutiérrez-Sánchez et al. 2024; Haugan 2021). A recent systematic review conducted by Gutiérrez-Sánchez et al. (2024) demonstrated that individuals who perceived a supportive network to be available frequently reported receiving assistance from social networks during stressful situations within the context of caregiving. Additionally, Krok et al. (2024) reported that individuals with cancer who perceived social support from their networks were more likely to receive assistance, enabling them to manage their diagnosis more effectively while experiencing reduced anxiety and isolation. Therefore, both perceived and received social support can have a positive impact on an individual’s well-being, reducing the sense of isolation in stressful situations.

Peer support is among the most prevalent forms of social support, provided by family members, friends, UCCs with first-hand experience in cancer caregiving, and trained volunteers within the community (Adamakidou et al. 2023; Blanco et al. 2023; De Maria et al. 2020). Like social support, peer support can reduce the impact of stressful situations by shaping individuals’ responses to these conditions, offering coping mechanisms, providing information and guidance, and assisting in addressing the challenges associated with cancer caregiving (Dennis 2003; Husted et al. 2022; Skirbekk et al. 2018).

However, despite the positive impact of social and peer support on UCCs, the high demand for care required by individuals with cancer results in numerous unmet supportive needs for UCCs (Molassiotis and Wang 2022). While several studies have focused on the supportive needs of individuals with cancer and their UCCs in the early stages of cancer, including emotional distress and anxiety about cancer recurrence (Lambert et al. 2012; Miroševič et al. 2019), limited research has explored the unmet needs of UCCs residing in rural and remote areas. Stiller et al. (2021) also note that UCCs living in rural and remote areas require additional support in relation to finances, transportation, cancer symptom management, as well as their own physical and emotional well-being from their social networks. As a result, UCCs who reside in rural and remote areas are more likely to experience a lower quality of life, poorer physical health, higher burden of care, and greater financial issues than their urban counterparts (O’Connor et al. 2018; Xu et al. 2021). While it is known that UCCs may face unique challenges which can be exacerbated by rural and remote contexts, no systematic review has been conducted to date to explore perceived and/or received peer support needs of UCCs residing in rural and remote areas with a specific focus on informational, practical, and emotional needs.

## Aim

This systematic review aims to explore the perceived and/or received peer support needs of UCCs residing in rural and remote areas with a specific focus on informational, practical, and emotional needs.

## Methods

This systematic review was undertaken following the 2020, 27-Item Preferred Reporting Items for Systematic Reviews and Meta-Analyses (PRISMA) reporting (Page et al. 2021). To assess the quality of the articles, the Mixed Methods Appraisal Tool (MMAT) was used to encompass all study designs, including qualitative, quantitative, and mixed-method studies (Hong et al. 2018). The systematic review was registered on PROSPERO CRD42024544502.

### Eligibility criteria

Primary research studies of any methodology were included if they focused on: (1) UCCs (such as family members, parents, siblings, partners, and adult children) who provided care to an adult family member or a friend diagnosed with advanced terminal cancer and lived in remote and rural areas; (2) for this review UCCs were considered to be adults and over 18 years old; (3) primary studies were included if they explored UCCs perceived and/or received support provided by their peers, such as other UCCs, family members, friends and trained volunteers; (4) were studies published in English from 2004 to 2024, as the earliest research on online peer support emerged post 2004.

### Exclusion criteria

The research was excluded if it related to: (1) UCCs of children with cancer due to the complexity of additional needs that UCCs may require in managing the potential loss of a child; (2) grey literature; (3) conference papers, abstracts, discussions, reports, chapter books, guidelines, protocols, editorials, and opinion papers; (4) protocols, and tool validation studies.

### Search strategy

An advanced search of databases CINAHL, ScienceDirect, PubMed/ Medline (via Ovid), ProQuest, Web of Science, Scopus, and Informit between 2004 and 2024 was conducted to identify studies that explored the perceived and/or received peer support in meeting informational, practical, and emotional needs of UCCs residing in rural and remote areas. The following concepts were included in the initial key concepts: “Peer*” OR “Support*” OR “Peer group” OR “social support” AND “psycho-oncology” OR “cancer” OR “neoplasms,” AND “Caregiv*” OR “family” AND “terminally ill” OR “hospices” AND “rural population.” The completed search strategies (inclusive of equivalent concepts) are presented in *the supplementary supporting material.*

### Study selection

After retrieving relevant studies, all duplicates were removed through Endnote 20. Studies were then imported into the Joanna Briggs Institute Unified Management, Assessment and Review of Information (JBI SUMARI) to facilitate the selection process (Piper 2019). MK and BV assessed titles and abstracts against the eligibility criteria independently. Following this, MK and BV reviewed the selected full-text articles. Any conflicts were resolved by the third and fourth authors, SS and LE, to reach a consensus.

### Quality assessment appraisal checklist

The MMAT was employed to assess the quality of the articles (Hong et al. 2018). The MMAT tool appraises the methodological quality of studies, including qualitative studies, mixed-method studies, and quantitative studies through “Yes,” “No,” or “Cannot tell” responses. All types of studies were initially assessed by identifying clear research questions and congruency with collected data. The tool consists of 5 additional questions based on specific study designs and assesses aspects of methodological approaches focused on data collection methods, alignment between methodology and aims, data analytic techniques, results and rigour, and validity. The authors of the MMAT tool recommend avoiding using a summative numerical score since a single score does not provide comprehensive information on which aspects of the study might be unclear. As a result, Hong et al. (2018) advise presenting a detailed rating from each criterion to ensure the methodological quality of each included study is well-informed. For the purposes of this study, 3 authors appraised the studies independently, and any conflicts were resolved through discussion (Appendix A).

### Data extraction

Data were extracted using the JBI SUMARI data extraction method (Piper 2019). The characteristics of each article, including the author(s), year, country, study design, sample size, recruitment methods, data collection tools, data analysis tools, and findings, were extracted (Appendix B).

### Data synthesis

Due to the non-homogenous of the included studies, a thematic narrative analysis was used for data synthesis (Popay et al. 2006). The stages of analysis included familiarisation, generating initial codes, searching for themes, and reviewing and defining the themes (Braun and Clarke 2006). Coding was undertaken by MK and BV, and a codebook was developed after discussion with senior researchers LE and SS to reach consensus. Examples of initial codes included: limited post-bereavement support, peer support improved self-confidence, peer support as a bridge to healthcare services, limited professional healthcare services, lack of reliability in peer support, and UCCs were neglected by their peers. The codes were then grouped into similar themes by MK and BV. Themes were constantly developed and shared with the third and fourth authors, SS and LE, in regular meetings to reach a consensus, to ensure methodological rigour and reduce any potential bias.

## Results

### Study characteristics

**Figure 1.**
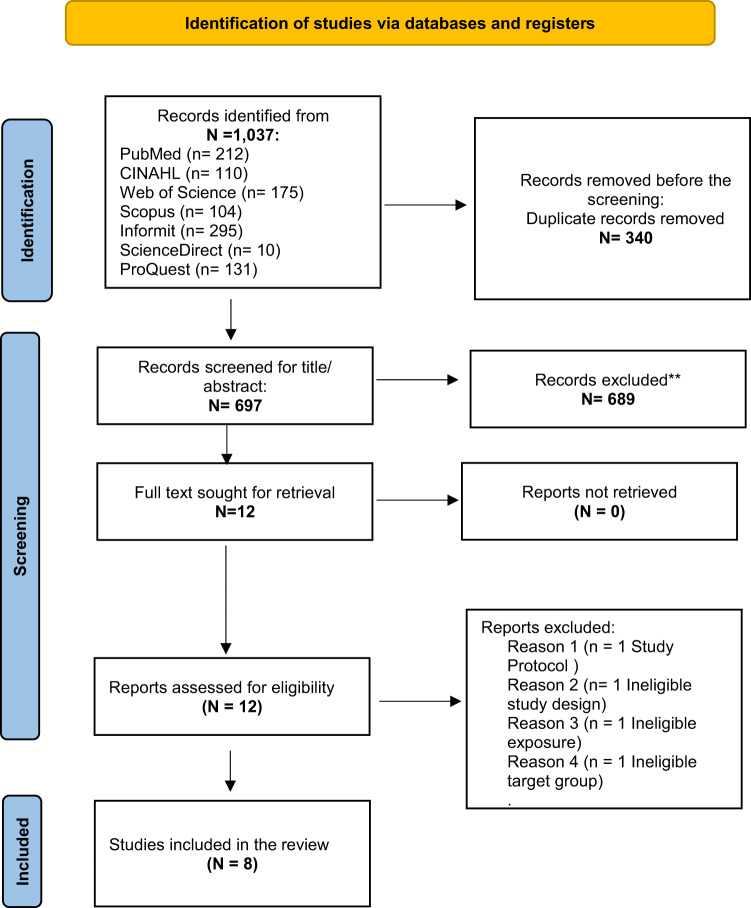
Preferred Reporting Items for Systematic Reviews and Meta-Analyses (PRISMA) flow diagram.

A total of 1037 primary studies were extracted from the 7 databases. After removing 340 duplicated articles, MK and BV independently screened the titles and abstracts of 697 studies. There was a total of 8 studies after 12 full-text screenings. Four qualitative studies (Jack et al. 2011; Lockie et al. 2010; Perera et al. 2021; Winter et al. 2023), 2 mixed-method (Hudson et al. 2008; Larocque et al. 2024), 1 cross-sectional study (Brazil et al. 2013), and a prospective survey (Ervik et al. 2020) were included. The final search results are detailed in the PRISMA flow diagram (Figure 1).

Following data synthesis, 4 main themes were identified regarding the perceived and/or received peer support on meeting UCCs’ unmet needs residing in rural and remote areas. The 4 themes are: (1) Emotional, practical, and informational unmet needs; (2) Lack of peer support on the physical and emotional well-being of UCCs in remote and rural areas; (3) Lack of supportive services in remote and rural areas, and (4) Access to flexible peer support. A summary of the findings of the included studies is in Appendix B.

### Emotional, practical, and informational unmet needs

Six of the eight included studies focused on the emotional, practical, and informational needs of UCCs (Hudson et al. 2008; Jack et al. 2011; Ervik et al. 2020; Perera et al. 2021; Winter et al. 2023; Larocque et al. 2024). The emotional needs perceived by UCCs were a lack of bereavement support (Jack et al. 2011; Perera et al. 2021; Winter et al. 2023; Larocque et al. 2024), practical needs associated with symptom management such as constipation, nausea, and vomiting commonly experienced among individuals who are living with advanced cancer (Perera et al. 2021; Larocque et al. 2024), medication administration (Hudson et al. 2008; Jack et al. 2011), pain management (Hudson et al. 2008; Jack et al. 2011), and informational needs related to the process of a natural death and end-of-life care (Perera et al. 2021; Larocque et al. 2024).

UCCs stated that further emotional support was needed when cancer reached advanced stages among their loved ones, and also during bereavement, by both counselling services and their peers (Jack et al. 2011; Perera et al. 2021; Winter et al. 2023). The need for emotional support was due to the support provided by both peers and healthcare services being intermittent and UCCs perceiving this to be insufficient, as they dealt with the health deterioration of their loved ones on their own and without appropriate support (Winter et al. 2023; Larocque et al. 2024).

UCCs also felt they needed to be adequately trained to undertake the practical skills, such as symptom management and medication administration for the individuals they were caring for. UCCs stated that due to a lack of regular training in symptom management (Perera et al. 2021), and the burden of responsibilities undertaken by UCCs (Perera et al. 2021; Larocque et al. 2024), they found it difficult and felt overwhelmed to perform such tasks in a short period of time (Perera et al. 2021). However, UCCs acknowledged the benefit of receiving regular training sessions in the form of structured programs led by healthcare staff and peers (Hudson et al. 2008) or trained volunteers (Jack et al. 2011). Receiving training on medication administration led to patients being encouraged to adhere to their treatment (Jack et al. 2011), and enhanced UCCs’ preparedness (*p* < 0.001) and competence (*p*-value = 0.01) among 54 participants (Hudson et al. 2008) in performing tasks independently.

As cancer approaches the terminal stages, pain management becomes a primary area of concern for UCCs. As a result, UCCs felt they lacked the necessary skills in effective pain assessment and management (Hudson et al. 2008; Jack et al. 2011). UCCs who had not received adequate support from oncology healthcare staff experienced uncertainty about administering the correct doses of analgesics to their loved ones (Jack et al. 2011). However, Hudson et al. (2008) reported that UCCs who participated in peer support group sessions not only learned analgesic medication administration, but also benefited from non-pharmacological pain management such as relaxation techniques and massage, leading to an improvement in confidence associated with the provision of pain management.

The need for information, particularly associated with death and dying, was common among the studies. For example, Larocque et al. (2024) found that, similar to Perera et al. (2021), UCCs wanted to learn the signs of the dying process and how to provide end-of-life care. Moreover, over 40% of UCCs stated that they needed information on what to expect regarding their loved one’s cancer trajectory (Larocque et al. 2024). The need for information regarding the process of death was due to individuals with cancer being more likely to die at home, where there is limited access to healthcare services (Perera et al. 2021; Larocque et al. 2024). While the study conducted by Ervik et al. (2020) reported that 58% of 181 UCCs needed information regarding cancer caregiving from their peers, the findings did not clarify the type of information UCCs were seeking.

### Lack of peer support on the physical and emotional well-being of UCCs in rural areas

Five of the eight papers reported that UCCs physical and emotional well-being and health status were adversely impacted due to the lack of peer support (Lockie et al. 2010; Jack et al. 2011; Perera et al. 2021; Winter et al. 2023; Larocque et al. 2024). Lack of support from peers, trained volunteers, friends, and other family members led to chronic fatigue, sleep deprivation (Lockie et al. 2010; Larocque et al. 2024; Winter et al. 2023), increased emotional distress and anxiety (Perera et al. 2021; Winter et al. 2023), as well as a sense of neglect and isolation (Winter et al. 2023; Larocque et al. 2024) in UCCs residing in remote and rural areas.

Two studies reported that UCCs felt they were burdened by cancer caregiving responsibilities on their own (Winter et al. 2023; Larocque et al. 2024), which led to sleep deprivation (Larocque et al. 2024) and fatigue (Winter et al. 2023; Larocque et al. 2024). The lack of sleep and chronic fatigue were due to UCCs being constantly concerned about their loved one’s condition (Winter et al. 2023), and the potential for deterioration during the night (Larocque et al. 2024). The limited access to volunteers for commuting to healthcare services led UCCs to drive long distances without consistent breaks, which was also associated with sleep deprivation and fatigue among UCCs (Lockie et al. 2010).

Findings from Perera et al. (2021) and Larocque et al. (2024) indicated that UCCs with limited support from peers had fewer opportunities to express their feelings regarding the emotional burden and frustration arising from caregiving, which resulted in an increase in anxiety and emotional distress. The emotional distress was exacerbated during the imminent death of their loved ones (Jack et al. 2011; Larocque et al. 2024). Additionally, UCCs experienced a sense of isolation and neglect during the bereavement period as emotional support from peers and counselling services ceased following the passing of their loved ones with cancer (Winter et al. 2023; Larocque et al. 2024).

### Lack of supportive services in remote and rural areas

Six of the eight papers discussed a lack of peer support groups (Ervik et al. 2020; Perera et al. 2021; Winter et al. 2023), and oncology services, including professionally trained oncology staff and respite care (Jack et al. 2011; Brazil et al. 2013; Perera et al. 2021; Winter et al. 2023; Larocque et al. 2024) for UCCs to seek informational, practical, and emotional support needs in remote and rural areas. In terms of oncology services, the literature found that there was a shortage of oncology specialists in rural and remote areas, and in 1 study leading the majority of UCCs (65.1% of 100 participants) to primarily rely on family physicians or general emergency rooms for either urgent situations or symptom management (Brazil et al. 2013). The lack of supportive services extends to respite care in rural and remote areas where there is a lack of oncology healthcare staff (Brazil et al. 2013; Perera et al. 2021; Larocque et al. 2024). Brazil et al. (2013) reported that from 100 participants, approximately 11% of UCCs residing in rural and remote areas benefited from respite services. However, peers, including trained volunteers, played a crucial role in referring individuals with advanced cancer and their UCCs to hospice care by locating them, particularly for those residing “deep in the village” in very remote and rural areas, which are difficult for the hospice team to reach (Jack et al. 2011). As a result, UCCs stated the need for reliable respite care services to take a break from caregiving and to manage their own physical and emotional well-being while ensuring their loved ones are well-cared for by oncology-trained healthcare professionals (Perera et al. 2021; Larocque et al. 2024). Moreover, the need for respite care increased during the COVID-19 pandemic due to extended lockdowns (Winter et al. 2023).

Multiple studies found that not only are oncology services limited in remote and rural areas for UCCs to seek support, but there are also limited local community resources, such as peer support groups, for UCCs to seek support from (Ervik et al. 2020; Perera et al. 2021; Winter et al. 2023). UCCs reported a need for peer support groups tailored to rural contexts and advanced-stage cancer (Perera et al. 2021; Winter et al. 2023). Similarly, Ervik et al. (2020) added that from 181 participants, over 85% of UCCs requested to have access to peer support, particularly tailored to their rural context. The need for access to tailored peer support groups was due to the unique needs of individuals during advanced stages of cancer, and the challenges UCCs face, such as distance to nearest supportive services (Ervik et al. 2020; Perera et al. 2021; Winter et al. 2023). Perera et al. (2021) also suggested that tailored peer support for specific groups, such as Lesbian, Gay, Bisexual, Transgender (LGBT, non-binary sexuality) or non-English Speakers, exclusively in rural and remote areas, can help to address the unique needs of UCCs who are providing care for individuals at an advanced stage.

### Access to flexible peer support resources

Two studies reported that UCCs can benefit from online resources and telephone conversations along with face-to-face sessions for UCCs who are caring for their loved ones at advanced stages of cancer at home in rural and remote areas (Perera et al. 2021; Winter et al. 2023). Winter et al. (2023) reported that UCCs would be supported in meeting their needs through telephone conversations and online chats with their peers, as they were not able to leave their loved ones “unattended.” However, despite the accessibility to substantial information on online platforms regarding cancer caregiving, UCCs may find it difficult to use such resources due to limited computer literacy or consider them to lack quality (Perera et al. 2021). Conversely, Winter et al. (2023) and Perera et al. (2021) emphasised that as online platforms became more “user-friendly” in the post-COVID-19 period, the engagement through watching shared videos, downloading informational resources, and interacting with peers through chat functions among UCCs increased.

Despite the benefits of online peer support, 4 papers reported on the lack of access to flexible support groups that can be accessed at any time and from anywhere by UCCs. There was limited access to peer support groups located in rural and remote areas for UCCs who provided care for individuals with advanced cancer (Brazil et al. 2013; Ervik et al. 2020). As a result, 89% of UCCs requested to have easy access to peer support groups located in rural and remote areas to meet their peers in person on a regular basis (Ervik et al. 2020) to share their experiences (Ervik et al. 2020; Perera et al. 2021; Winter et al. 2023) and to acknowledge their role as a caregiver (Ervik et al. 2020; Winter et al. 2023).

By attending face-to-face peer support sessions, UCCs were more likely to build a social network and receive a sense of acceptance as UCCs residing in remote and rural areas (Ervik et al. 2020; Winter et al. 2023). Among 181 participants, approximately 54% of UCCs stated that the interaction between peers can close the gap left by a shortage of healthcare staff in rural and remote areas, where it might be difficult to access informational, practical, and emotional support (Ervik et al. 2020).

## Discussion

This systematic review aimed to explore the perceived and/or received peer support needs of UCCs residing in rural and remote areas with a specific focus on informational, practical, and emotional needs. Key findings indicated that UCCs face multifaceted unmet needs throughout the advanced cancer stage. The findings indicated that due to the lack of supportive services, including peer support and oncology services, UCCs well-being was negatively impacted. Moreover, the results emphasised that it is crucial to have easy access to peer support for UCCs that is tailored to their circumstances and rural context to maximise the benefits.

The findings reflected that additional support is required from peers for UCCs to perform advanced tasks during end-of-life care, which can be challenging to manage on their own, especially when it might be beyond their knowledge and capabilities (Bijnsdorp et al. 2020; Too et al. 2023). The findings in similar studies highlighted that the need for caregiving support does not necessarily end with an individual’s death, but it also continues until UCCs have effectively coped with grief (Wen et al. 2019; Ploukou et al. 2023) to prevent complicated grief (Nielsen et al. 2017).

Providing care without support from peers or other social networks negatively impacts the well-being of UCCs living in remote and rural areas. Similar to this review, findings by Johnston et al. (2024) indicated that UCCs providing caregiving tasks alone, with limited access to peer support, are associated with negative health effects such as lack of sleep and fatigue. Conversely, Breuning et al. (2024) reported that UCCs who engaged in peer support groups were less likely to be emotionally distressed and anxious than those who were isolated.

Previous studies support the finding in this systematic review that there is a need for respite care for UCCs residing in remote and rural areas (Nankervis et al. 2011; Ugalde et al. 2019). Furthermore, Allicock et al. (2017) stated that peer support groups tailored to the rural context and advanced stages of cancer can provide UCCs with peers who have a firsthand understanding of the local community, a shared experience of cancer caregiving, and who are often more accessible and easier to communicate with compared to healthcare staff (Gunn et al. 2022). Although peers can help to address the gap in healthcare services in rural areas, they cannot substitute healthcare professionals (Kirby et al. 2016). Therefore, the presence of both peers and healthcare staff is essential to provide holistic support for UCCs residing in remote and rural areas.

While face-to-face peer support has been shown to help UCCs feel more prepared for their caregiving role through shared experiences (Kinnane et al. 2011), online peer support groups can be an alternative in remote and rural areas where it is challenging to have access to face-to-face support groups (Benson et al. 2020). Similar to this review, Rodler et al. (2021) found that older adults with limited computer literacy became more likely to join online peer groups when interfaces were simplified. However, McLoughlin et al. (2023) and Gunn et al. (2022) both highlight that some caregivers still face challenges due to low confidence in using technology or poor digital infrastructure, especially in rural and remote areas. Attrill and Jalil (2011) also suggest that while online peer support provides rapid access to information, using social media lacks in quality and cannot be trusted in some circumstances. Therefore, combining face-to-face and telecommunication-based peer support may be the most effective approach to overcome access barriers in remote and rural regions (Lauckner and Hutchinson 2016).

### Implications for practice

The findings of this systematic review indicate that closer collaboration is required between UCCs and healthcare professionals to establish and maintain reliable peer support groups for unpaid caregivers of individuals with advanced cancer. These peer support groups should aim to address UCCs unmet needs in a specific stage of cancer within a rural context and in a readily accessible way. While the accessibility to peer support should be facilitated by combining face-to-face sessions with ongoing online or telephone-based interactions, training should be provided for UCCs who have limited technology literacy to benefit from such support in rural and remote areas.

### Recommendations for future studies

Future studies are recommended to focus on developing a comprehensive practical care support model for UCCs to address their current needs within the context of rural and remote areas. It is also recommended that potential methods of delivering support be considered to ensure an effective, practical, and accessible support system in these areas. It is worth noting that cultural, socioeconomic status, and healthcare delivery framework factors should be considered to ensure models of care are adaptable and feasible in different rural contexts. Additionally, it is also recommended that future studies also consider cultural diversity among UCCs to reflect how cultural influences can affect peer support needs.

### Strengths and limitations

To our knowledge, this is the first systematic review conducted to explore the perceived and/or received peer support needs of UCCs residing in rural and remote areas with a specific focus on informational, practical, and emotional needs. It has been identified that both healthcare staff and social networks are integral parts of a supportive system for UCCs. Previous reviews have mainly focused on the unmet needs of UCCs; however, the needs of those who were providing care for individuals with advanced cancer in rural and remote areas were neglected. A limitation of this systematic review is that the number of primary studies included was 8, which hindered the authors from exploring cultural diversity and reflecting differences across different sociodemographic groups.

## Conclusion

This systematic review underscores perceived and/or received peer support in meeting UCCs informational, emotional, and practical needs residing in rural and remote areas. Peer support is valued in fostering a sense of acceptance and sharing similar experiences and meeting the complex cancer caregiving needs of UCCs during the advanced stage of cancer. It was also found that the significance of collaboration between healthcare systems and local communities to provide support, and how a lack of peer support may negatively impact UCCs’ overall well-being in rural and remote areas. Furthermore, the review highlighted the importance of combining face-to-face sessions, telephone conversations, and online support groups to facilitate the delivery of peer support.

## Appendix A: MMAT Quality Appraisal Checklist for Quantitative Descriptive Studies

**Table:**

Studies	S1	S2	1.1	1.2	1.3	1.4	1.5
(Jack et al. 2011)	✓	✓	✓	✓	✓	✓	✓
	✓	✓	✓	✓	✓	✓	✓
(Lockie et al. 2010)	✓	✓	✓	✓	✓	✓	✓
	✓	✓	✓	✓	✓	✓	✓
(Perera et al. 2021)	✓	✓	✓	✓	✓	✓	✓
	✓	✓	✓	✓	✓	✓	✓
(Winter et al. 2023)	✓	✓	✓	✓	✓	✓	✓
	✓	✓	✓	✓	✓	✓	✓

✓ (Yes), ✗ (No), ? (Cannot tell)

S1. Are there clear research questions?

S2. Do the collected data allow to address the research questions?

1.1. Is the qualitative approach appropriate to answer the research question?

1.2. Are the qualitative data collection methods adequate to address the research question?

1.3. Are the findings adequately derived from the data?

1.4. Is the interpretation of results sufficiently substantiated by data?

1.5. Is there coherence between qualitative data sources, collection, analysis, and interpretation?

**Table:**

Studies	S1	S2	5.1	5.2	5.3	5.4	5.5
(Hudson et al. 2008)	✓	✓	✓	✓	✓	?	✓
	✓	✓	✓	✓	✓	?	✓
(Larocque et al. 2024)	✓	✓	✓	✓	✓	?	✓
	✓	✓	✓	✓	✓	?	✓

✓ (Yes), ✗(No), ? (Cannot tell)

S1. Are there clear research questions?

S2. Do the collected data allow to address the research questions?

5.1. Is there an adequate rationale for using a mixed methods design to address the research question?

5.2. Are the different components of the study effectively integrated to answer the research question?

5.3. Are the outputs of the integration of qualitative and quantitative components adequately interpreted?

5.4. Are divergences and inconsistencies between quantitative and qualitative results adequately addressed?

5.5. Do the different components of the study adhere to the quality criteria of each tradition of the methods involved?

**Table:**

Studies	S1	S2	4.1	4.2	4.3	4.4	4.5
(Ervik et al. 2020)	✓	✓	✓	✓	✓	?	✓
	✓	✓	✓	✓	✓	?	✓

✓ (Yes), ✗ (No), ? (Cannot tell)

S1. Are there clear research questions?

S2. Do the collected data allow to address the research questions?

4.1. Is the sampling strategy relevant to address the research question?

4.2. Is the sample representative of the target population?

4.3. Are the measurements appropriate?

4.4. Is the risk of nonresponse bias low?

4.5. Is the statistical analysis appropriate to answer the research question?

**Table:**

Studies		S1	S2	3.1	3.2	3.3	3.4	3.5
(Brazil et al. 2013)	✓		✓	✓	✓	✓	?	✓
	✓		✓	✓	✓	✓	?	✓

✓ (Yes), ✗ (No), ? (Cannot tell)

S1. Are there clear research questions?

S2. Do the collected data allow to address the research questions?

3.1. Are the participants representative of the target population?

3.2. Are measurements appropriate regarding both the outcome and intervention (or exposure)?

3.3. Are there complete outcome data?

3.4. Are the confounders accounted for in the design and analysis?

3.5. During the study period, was the intervention administered (or exposure occurred) as intended?

## Appendix B

**Table:**

*Authors (year)*	*Country*	*Study design*	*Sample size*	*Recruitment methods*
Brazil et al. (2013)	Canada	Cross-sectional study	100	Healthcare databases in rural areas, contacting potential participants through telephone
Ervik et al. (2020)	Norway	Prospective survey	181	Advertising and referrals from cancer support center
Hudson et al. (2008)	Australia	Mixed-method study	74	Home-based palliative care services
Jack et al. (2011)	Uganda	Qualitative study	21	Community Hospice and volunteer workers’ networks
Larocque et al. (2024)	Canada	Parallel mixed-method study	44	Home-visiting service hospice center
Lockie et al. (2010)	Canada	Qualitative study	15	Regional cancer care centers and community outreach
Perera et al. (2021)	Australia	Qualitative study	20	Outpatient waiting rooms in rural hospitals
Winter et al. (2023)	Australia	Qualitative study	12	Advertising on websites, local newsletters, and social media, and promotion of the study by clinicians at health services.

**Table:**

*Authors (year)*	*Data collection tools*	*Data analysis tools*	*Findings*
Brazil et al. (2013)	CBS-EOLC, MSPSS, Health Service Use, and ECOG	Likelihood ratio test, and independent Student’s t-tests	UCCs experienced significant isolation due to limited rural healthcare services, highlighting the critical shortage of professional and supportive services.
Ervik et al. (2020)	A comprehensive prospective survey (no further details reported)	Spearman’s rank correlation, and X2 or Fisher’s exact test	UCCs highlighted peer interactions as valuable, improving their well-being and acceptance, and emphasized the importance of peer support complementing professional healthcare services.
Hudson et al. (2008)	Semi-structured interviews, facilitators’ journals, and self-report instruments: CCS, PCS, FIN, RCS, SSQ, BASC, LOT, and COMPET	Qualitative data: Thematic analysis. Quantitative data: Inferential and descriptive statistics using repeated measures ANOVAs	There were improvements in caregiver preparedness and competence. UCCs were feeling emotionally reassured, UCCs were validated by peer interactions and experienced improved confidence in caregiving tasks.
Jack et al. (2011)	Face-to-face semi-structured interviews	Thematic analysis	UCCs considered volunteers to play a crucial role for linking them to hospice care and offering emotional support, helping reduce feelings of isolation.
Larocque et al. (2024)	Telephone-based semi-structured interviews, DT, CSNAT	Qualitative data: constant comparative thematic analysis. Quantitative data: the Mann-Whitney U Test, Spearman’s correlation	UCCs stated emotional distress and inadequate informational and practical support; such as symptom management and end-of-life care.
Lockie et al. (2010)	Face-to-face or telephone-based in-depth semi-structured interviews.	Thematic analysis	UCCs reported sleep deprivation and fatigue from commuting and described the isolation.
Perera et al. (2021)	Telephone-based or face-to-face semi-structured interviews, focus groups.	Thematic analysis	UCCs expressed doubts regarding the reliability of peer-sourced information; and highlighted the need for trustworthy, professional resources to ensure confidence in caregiving.
Winter et al. (2023)	Telephone-based semi-structured interviews	Thematic analysis	UCCs experienced isolation, emotional neglect, and abandonment following the death of their loved ones, highlighting a substantial gap in post-bereavement support from their peers and professional supportive services.

**Note**: Caregiver’s Burden Scale in End-of-Life Care = CBS-EOLC; Multidimensional Scale of Perceived Social Support = MSPSS; and Eastern Collaborative Oncology Group = ECOG; Caregiver competence scale = CCS; Preparedness for caregiving scale = PCS; Family inventory of need = FIN; Rewards for caregiving scale = RCS; Social Support Questionnaire = SSQ; Brief assessment scale for caregivers = BASC; Life orientation test = LOT; COMPET = competence; Distress Thermometer short screening tool = DT; Carer Support Needs Assessment Tool = CSNAT.
